# Prognostic Protein Biomarker Screening for Thyroid Carcinoma Based on Cancer Proteomics Profiles

**DOI:** 10.3390/biomedicines12092066

**Published:** 2024-09-10

**Authors:** Pu Xie, Qinglei Yin, Shu Wang, Dalong Song

**Affiliations:** 1 Department of Endocrine and Metabolic Diseases, Shanghai Institute of Endocrine and Metabolic Diseases, Ruijin Hospital, Shanghai Jiao Tong University School of Medicine, Shanghai 200025, China; xiepu1997@163.com; 2 Shanghai National Clinical Research Center for Metabolic Diseases, Key Laboratory for Endocrine and Metabolic Diseases of the National Health Commission of the PR China, Shanghai Key Laboratory for Endocrine Tumor, Ruijin Hospital, Shanghai Jiao Tong University School of Medicine, Shanghai 200025, China; 3 Guangdong Geriatric Institute, Guangdong Provincial People’s Hospital (Guangdong Academy of Medical Sciences), Southern Medical University, Guangzhou 510000, China; yinqinglei128@163.com; 4 Reproductive Medicine Center, Department of Obstetrics and Gynecology, Guangdong Provincial People’s Hospital (Guangdong Academy of Medical Sciences), Southern Medical University, Guangzhou 510000, China

**Keywords:** thyroid carcinoma, TCPA, overall survival, TMB

## Abstract

Thyroid carcinoma (THCA) ranks among the most prevalent cancers globally. Integrating advanced genomic and proteomic analyses to construct a protein-based prognostic model promises to identify effective biomarkers and explore new therapeutic avenues. In this study, proteomic data from The Cancer Proteomics Atlas (TCPA) and clinical data from The Cancer Genome Atlas (TCGA) were utilized. Using Kaplan–Meier, Cox regression, and LASSO penalized Cox analyses, we developed a prognostic risk model comprising 13 proteins (S100A4, PAI1, IGFBP2, RICTOR, B7-H3, COLLAGENVI, PAR, SNAIL, FAK, Connexin-43, Rheb, EVI1, and P90RSK_pT359S363). The protein prognostic model was validated as an independent predictor of survival time in THCA patients, based on risk curves, survival analysis, receiver operating characteristic curves and independent prognostic analysis. Additionally, we explored the immune cell infiltration and tumor mutational burden (TMB) related to these features. Notably, our study proved a novel approach for predicting treatment responses in THCA patients, including those undergoing chemotherapy and targeted therapy.

## 1. Introduction

Thyroid carcinoma (THCA) is one of the most common neoplastic diseases [1] and is expected to become the fourth leading type of cancer in the world [2]. In the USA, the incidence of THCA has increased rapidly from 4.56 cases per 100,000 person-years to 14.41 cases in the past four decades [3]. Risk factors of THCA include radiation exposure at a young age, excess body weight, hormonal exposures, certain environmental pollutants, and family history [4]. Most patients with THCA can be treated by surgery or radioactive iodine therapy followed by endocrine suppression [5]. Nevertheless, the mortality rate of advanced thyroid cancer remains a significant concern. Reports indicate an increased incidence of more advanced forms and higher morbidity associated with thyroid carcinoma [6,7]. Thus, the identification of novel biomarkers for prognosis and drug screening in THCA, providing additional strategies for survival and optimizing treatments, is of great significance.

The pathogenesis of THCA is not fully understood. With the development of high-throughput technologies, recent decades have witnessed great improvements in cancer genomics [8]. Using a multi-omics approach in recent years, the characteristics of THCA have been extensively studied in the aspects of genomics, transcriptomics, and proteomics [9]. With the application of proteomics, mass spectrometry-based proteomics enable the reclassification of cancer, which may affect prognosis and guide clinical decision-making [10]. Recently, prognostic signatures from public databases have garnered significant attention and demonstrated potentials for predicting outcomes in THCA patients. However, these studies were mainly focused on transcriptomic profiles with little known about proteome modulation and protein function. Previous studies have shown that, in many cases, transcript levels are insufficient to predict protein levels [11] and the average correlation between mRNA expression and protein expression is below 0.5 [12,13]. Therefore, prognostic signatures based on proteomic level will be crucial.

The application of advanced proteomics technologies and bioinformatics approaches to THCA research could offer unprecedented insight into cancer biology and treatment [14]. In our study, we determined a protein-associated prognostic signature from The Cancer Proteome Atlas (TCPA) THCA cohort. A risk model was thus established for predicting THCA patients’ prognosis. To validate the prognostic signature, we investigated its efficiency and accuracy using both training and testing sets. We also explored the association of this signature with immune cell infiltration and the tumor mutation burden (TMB) based on results from The Cancer Genome Atlas (TCGA). Importantly, our study proved a new approach for predicting the response to treatment, including chemotherapy and targeted therapy in THCA patients.

## 2. Materials and Methods

### 2.1. Data Collection

The protein expression data of THCA were retrieved from the TCPA (https://www.tcpaportal.org/tcpa/ (accessed on 18 July 2022)). The RNA-seq expression profile data (HTSeq-FPKM) of THCA patients were obtained from the TCGA data portal (http://portal.gdc.cancer.gov/cart (accessed on 18 July 2022)). Clinical information such as age, gender, survival time, survival status, and clinical stage was also downloaded from the TCGA data portal. Patients were randomly split into a training set and a testing set at a ratio of 5:5 using the “caret” packages. The distribution of age, gender, and clinical stage was similar between the two datasets (Supplementary Table S1). The training set was used to identify the prognostic signature and the testing set was used to validate its prognostic capability.

### 2.2. Construction of the Protein Prognostic Signature

To explore the possible protein in relation to prognosis for THCA patients, univariate Cox proportional hazard regression analysis was used to identify the relationship between prognostic proteins and OS in the training set, and *p* < 0.05 was considered to be statistically significant. After that, we used the “glmnet” packages [15,16], prognostic and selection operation (LASSO) to minimize overfitting and identify the most significant prognosis-related proteins. *p* < 0.05 was considered statistically significant and *p* < 0.01 was considered a significant difference. Risk score calculation formula: expression protein 1 × coefficient protein 1 + expression protein 2× coefficient protein 2 + … + expression protein *n* × coefficient protein n. A Kaplan–Meier plot was performed to compare the overall survival between two risk groups using the R package survival. The time-dependent receiver operating characteristic (ROC) curve was used to assess all the predictive values of the prognostic protein signature for overall survival using R package survivalROC. The AUC (Area Under ROC Curve) value of the ROC indicates that 0.5–0.7 is acceptable, 0.7–0.9 is good, and >0.9 is excellent.

### 2.3. Nomogram Construction and Calibration

The risk score and relevant clinical parameters such as age and stage were incorporated into the construction of a prognostic nomogram via an “rms” R package (version 6.2.0) to predict 1-, 3-, and 5-year OS of THCA patients in the TCPA cohort. We used a calibration plot comparing predicted and observed overall survival to evaluate the performance of the prognostic nomogram (method = “boot”, B = 1000).

### 2.4. DEPs Identification

We conducted a differential protein expression analysis in proteomic data. The R package limma (version 4.1.3) and Wilcox test were used to identify differentially expressed proteins (DEPs) between the high-risk and the low-risk group. Proteins with *p* < 0.05 were considered as DEPs.

### 2.5. Principal Component Analysis (PCA), GO and KEGG Analysis

PCA analysis was performed using the limma and scatterplot3d packages to explore the distribution of patients with different risk scores. To assess the potential biologic functions of differentially expressed proteins, Gene Ontology (GO) and Kyoto Encyclopedia of Genes and Genomes (KEGG) pathway enrichment analyses were performed by the cluster Profiler package in R. Functional categories with an adjusted *p* value < 0.05 were considered as significant pathways. To investigate the functional biological differences of the hub proteins, GO analysis was automatically completed and visualized by the Metascape [17].

### 2.6. Protein–Protein Interaction Network Construction

The common significant DEPs were mapped to the STRING database (http://www.string-db.org/ (accessed on 20 July 2022)), then set confidence > 0.7 to conduct the PPI network analysis, which were visualized by Cytoscape software (v3.9.1) [18]. Module analysis was performed to identify significant modules and explore the hub proteins using the Molecular Complex Detection (MCODE v2.0.0) [19], which is a plug-in of Cytoscape (MCODE score ≥ 6).

### 2.7. Gene Set Enrichment Analysis 

Gene set enrichment analysis (GSEA) was utilized to elucidate the molecular mechanisms. We divided the samples in the entire set into the high-risk and the low-risk groups based on scores from the signature and compared the KEGG pathway enrichment between the two groups. A *p*-value of <0.05 was considered significant.

### 2.8. Analysis of Tumor Infiltrating Immune Cells

We employed the CIBERSORT to evaluate the immune cell infiltration in each sample based on the RNA-seq data. With “CIBERSORT” (R package), we used the CIBERSORT algorithm to analyze gene expression data. Using the standard *p* < 0.05, we screened samples and calculated the percentage of 22 immune cells.

### 2.9. Analysis of Tumor Mutation Burden

The mutation data collected from TCGA were analyzed by R package maftools. The tumor mutation burden (TMB) was calculated using the formula: TMB = (total mutation/total covered bases) × 10^6^.

### 2.10. Immunotherapy Analysis

The Tumor Immune Dysfunction and Exclusion (TIDE) is a data-driven Web platform that combines large-scale omics data from 33,000 cases across 188 cohorts, 998 tumor samples from 12 immune checkpoint blockade (ICB) clinical studies, and eight clustered regularly interspaced short palindromic repeat (CRISPR) screens. TIDE aids in hypothesis generation and the optimization of immunological biomarkers. In this study, we used TIDE to evaluate immune-suppressive rejection scores and therapeutic responses to ICB.

### 2.11. Potential Chemotherapeutic Response

The response to common chemo drugs, such as temsirolimus, roscovitine, AZD6244, AKT inhibitor, and metformin, were predicted by the Genomics of Drug Sensitivity in Cancer (GDSC) (http://www.cancerrxgene.org/ (accessed on 20 July 2022)) to analyze the relationship between the signature and chemotherapeutic response. We used the R package *pRRophetic* to estimate and compare the half-maximal inhibitory concentration (IC50) between different risk groups.

### 2.12. Statistical Analysis

The differences in immune cells between the risk score were compared with the Wilcox test. The correlations between the expression of every protein in the signature and the expression of immune checkpoints were also calculated with Spearman rank correlation. All statistical analyses were performed with R software (version 4.1.3), and *p* < 0.05 was selected as statistically significant.

## 3. Results

### 3.1. Establishment of a Proteomic Prognostic Signature in Thyroid Carcinoma

**Figure 1:**
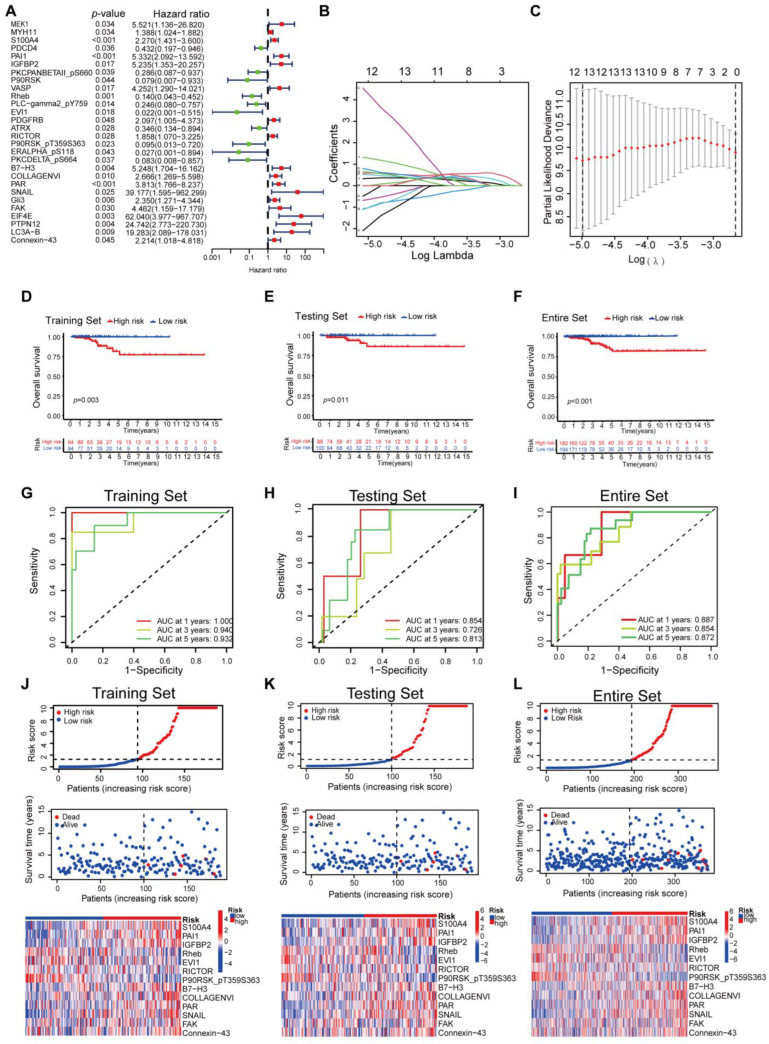
Establish of a Proteomic Prognostic Signature in Thyroid Carcinoma. (**A**) Forest plot from the univariate Cox regression analysis in the training set. (**B**) Least absolute shrinkage and selection operator (LASSO) regression of the OS-related proteins. (**C**) Cross-validation for tuning the parameter selection in the LASSO regression. (**D**–**F**) Kaplan–Meier plots of the training set (**D**), the testing set (**E**) and the entire set (**F**) stratified by the high-risk and low-risk groups based on the median risk score of the 13 signature proteins. (**G**–**I**) Time-dependent ROC curves showed the predictive efficiency of the risk scores in the training set (**G**), the testing set (**H**) and the entire set (**I**). (**J**–**L**) The risk score distribution, survival status of THCA cases, and protein expression profile of this prognostic signature in the training set (**J**), the testing set (**K**) and the entire set (**L**).

The whole dataset was split into a training set (*n* = 188) and a testing set (*n* = 188). The training set was used to develop the protein prognostic model, while the testing set and the entire dataset were used to validate the prognostic model. To screen prognosis-related proteins, univariate Cox regression analysis was conducted to identify proteins associated with prognosis. After univariate Cox analysis, 28 proteins met the criteria of *p* < 0.05 and were retained for further analysis. Among them, 18 proteins were associated with increased risk (HRs > 1), while the other 10 proteins were protective proteins (HRs < 1) (Figure 1A). Next, least absolute shrinkage and selection operator (LASSO) penalized Cox regression were applied to reduce the prognostic proteinsand a 13-protein signature was constructed according to the optimum λ value (Figure 1B,C, Supplementary Figure S1). The risk score could be calculated using the data in Table 1. Principal component analysis (PCA) demonstrated that the prognostic signature could effectively distinguish THCA patients into two groups (Supplementary Figure S2). We evaluated the risk model’s survival prediction performance using validation data. Samples from the training, testing, and entire datasets were categorized into high- and low-risk groups based on the median risk score. The individual risk score and survival status and the key protein expression profiles of patients are shown in Figure 1. Kaplan–Meier survival curves showed significantly worse overall survival (OS) for the high-risk group compared to the low-risk group (Figure 1D–F). Survival ROC curve analysis was also performed, and the area under the curve (AUC) at 1, 3, and 5 years was 1.000, 0.940, and 0.932, respectively, which verified the predictive value of the signature, and the AUC value of the test and the whole set also indicated a similar potential for predicting survival (Figure 1G–I). According to the risk graph, patient mortality appears to increase as the risk score increases (Figure 1J–L). In the high-risk group, S100A4, PAI1, IGFBP2, RICTOR, B7-H3, COLLAGENVI, PAR, SNAIL, FAK, and Connexin-43 proteins were upregulated, while Rheb, EVI1, and P90RSK_pT359S363 proteins were downregulated (Figure 1J–L). The risk curves and protein profile heatmap displayed features consistent with both the test set and the whole set (Figure 1J–L). These findings indicate that the protein profiles may serve as effective prognostic biomarkers for THCA patients.

### 3.2. Construction of Nomogram Based on the Protein Signature and Clinical Data

**Figure 2:**
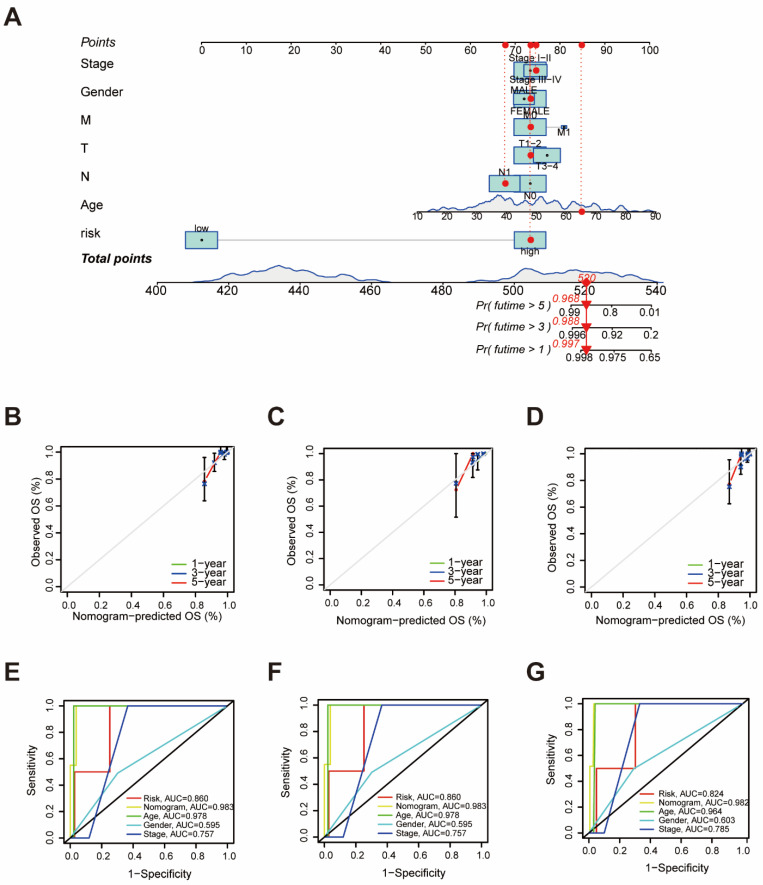
Construction of Nomogram Based on the Protein Signature and Clinical Data. (**A**) A nomogram based on the prognostic signature consisting of the risk score and clinical factors. (**B**–**D**) A calibration curve was plotted to show the alignment between the actual observed prognosis value and those predicted by the nomogram in the training set (**B**), the testing set (**C**), and the entire set (**D**). (**E**–**G**) ROC analysis of the proteomic signature and clinicopathological factors’ performance in the training set (**E**), the testing set (**F**), and the entire set (**G**).

To assess the independent prognostic force of the prognostic signature, both univariable and multivariable Cox proportional hazard regression models were applied in the training, the testing, and the entire sets (Supplementary Figure S3). The results demonstrated that the prognostic signature could serve as an independent predictor. A nomogram was then created using age, gender, TMN status, clinical stage, and risk-score levels to predict 1-, 3-, and 5-year overall survival in THCA patients (Figure 2A). The calibration curve presented desirable prediction of the nomogram for the 1-, 3-, and 5-year clinical outcomes (Figure 2B–D). Furthermore, it is notable that the AUC of the nomogram exceeded that of other characteristics (Figure 2E–G). These results suggest that the predictive nomogram was highly accurate for forecasting survival in THCA patients.

### 3.3. Clinical Relevance Assessment and Construction of the Protein Coexpression Network

**Figure 3:**
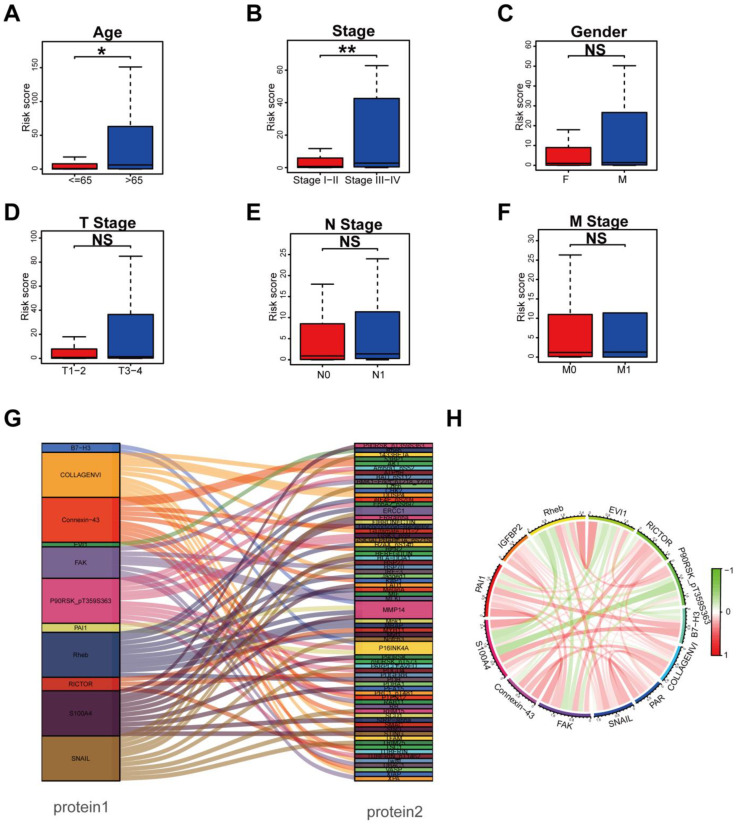
Clinical Relevance Assessment and Construction of the Protein Coexpression Network. (**A**–**F**) Comparison of the risk score in subgroups of patients with THCA. Patients with THCA were divided into age ≤ 65 and > 65 (* *p* < 0.05, ** *p* < 0.01, NS: no significance) (**A**), Stage I-II and Stage III-IV (**B**), different gender (**C**), T1–2 and T3–4 (**D**), N0 and N1 (**E**), M0 and M1 (**F**). (**G**) Sankey diagram of all proteins related to the 13 proteins in the TCPA database (correlation coefficient > 0.4) (*p* < 0.001). (**H**) The corelationship of 13 proteins in the prognostic signature.

In order to explore whether the prognostic signature participated in the development and progression of THCA, risk scores with clinical characteristics were compared. There were significant differences in the risk scores between different groups in age (*p* < 0.05) and TNM stage (*p* = 0.002) (Figure 3A,B), but no significant differences in gender, T stage, N stage, and M stage (Figure 3C–F). In addition, stratification analysis was further conducted to investigate the prognostic significance of THCA patients in subgroups. Our analyses suggested that a protein-based signature showed excellent performance in predicting outcome in age > 65 (*p* < 0.05), male (*p* < 0.05), female (*p* < 0.01), T3–4 stage (*p* < 0.01), N1 stage (*p* < 0.05), M0 stage (*p* < 0.01), Stage I–II (*p* < 0.05), and Stage III–IV (*p* < 0.01) (Supplementary Figure S4). Protein co-expression analysis for all 13 proteins was performed, and 69 proteins with correlation coefficients > 0.4 and a *p* value < 0.001 were identified (Figure 3G). The correlation of the proteins included in the prognostic signature is displayed in Figure 3H, in which Rheb and EVI1 showed the strongest positive correlation, while S100A4 and P90RSK_pT359S363 showed the strongest negative correlation. 

### 3.4. Functional Enrichment Analysis Based on Risk Model

**Figure 4:**
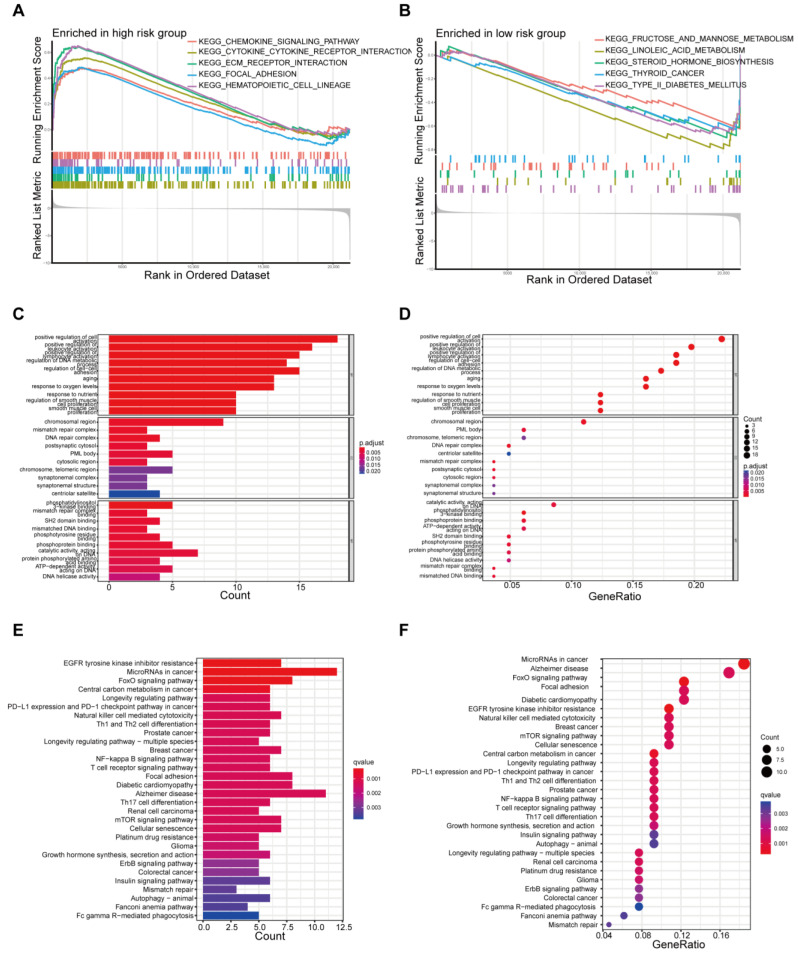
Functional Enrichment Analysis Based on Risk Model. (**A**,**B**) Gene set enrichment analysis (GSEA) in the high-risk group and the low-risk group. (**C**,**D**) Gene Ontology (GO) enrichment analysis of differentially expressed proteins. (**E**,**F**) Kyoto Encyclopedia of Genes and Genomes (KEGG) pathway analysis of differentially expressed proteins.

To identify the involved biological processes, we used GSEA to analyze protein expressions in THCA patients across different risk groups. In the high-risk group, representative KEGG pathways were chemokine signaling, cytokine–cytokine receptor interaction, ECM receptor interaction, focal adhesion, and the hematopoietic cell lineage pathway (Figure 4A). In the low-risk group, prominent KEGG pathways were fructose and mannose metabolism, linoleic acid metabolism, steroid hormone biosynthesis, and so on (Figure 4B). The differentially expressed proteins were further analyzed by GO and KEGG analysis. GO analysis revealed that primary functional categories in the biological processes (BP) were positive regulation of cell activation, regulation of DNA metabolic process and regulation of cell–cell adhesion. For cellular components (CCs), the major enriched GO terms were the chromosomal region, mismatch repair complex, and DNA repair complex. In terms of molecular functions (MFs), catalytic activity acting on DNA, phosphatidylinositol 3-kinase binding and phosphoprotein binding were enriched (Figure 4C,D, Supplementary Figure S5A,B). The KEGG pathway indicated that the differentially expressed proteins were mainly involved in EGFR tyrosine kinase inhibitor resistance, FoxO signaling central carbon metabolism in cancer, and the mTOR signaling pathway (Figure 4E,F, Supplementary Figure S5C,D).

To uncover the potential relationship between the DEPs, a PPI network that included 161 nodes and 256 edges was identified through the STRING tool. Then, the most significant modules were recognized by the MCODE plug-in of cytoscape. RBBP8 (CTIP), PMS2 (ATM), TP53BP1 (P53), MLH1 (MLH1), and MSH2 (MSH2) occupied the dominant position (Supplementary Figure S6A). GO analysis showed that the hub proteins were mainly engaged in response to stimulus and regulation of the biological process, metabolic process and immune system process (Supplementary Figure S6B).

### 3.5. Difference in Tumor-Infiltrating Immune Cells in Different Risk Groups

**Figure 5:**
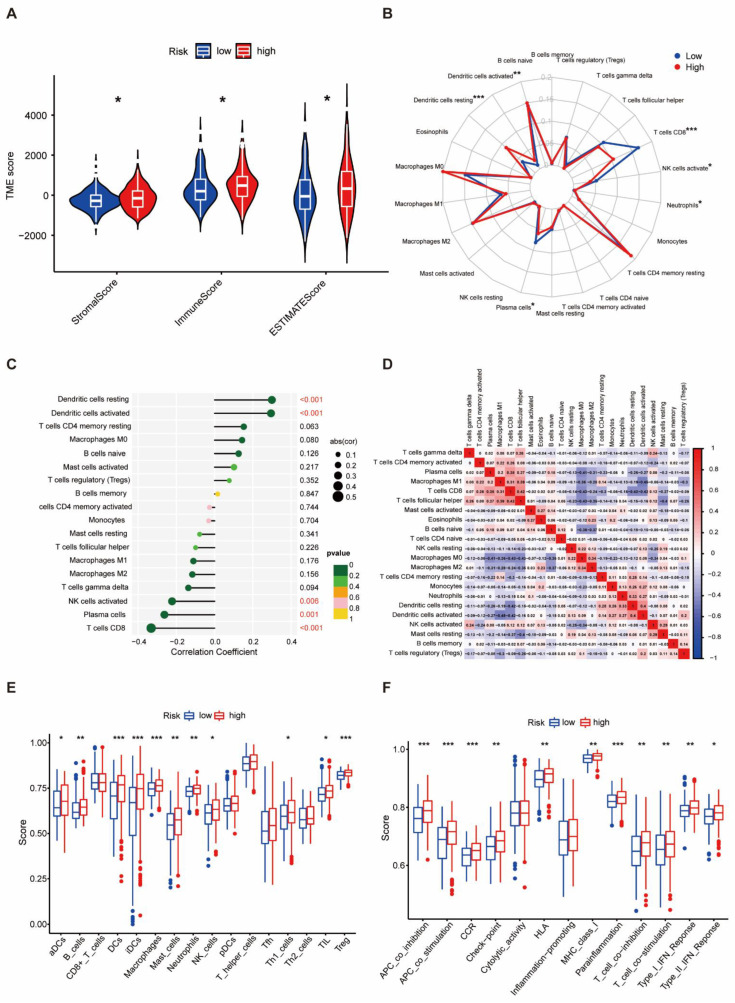
Difference in Tumor-Infiltrating Immune Cells in Different Risk Groups. (**A**) Comparison of the stromal score, immune score, and ESTIMATE score in the low- and the high-risk groups. (**B**) Radar map showing the scores of 22 immune cells in the low- and high-risk groups. (**C**) The correlation of risk score and the tumor-infiltrating immune cells. (**D**) The correlation of 22 immune cells in THCA. (**E**) Comparison of the enrichment scores of 16 types of immune cells between the low- and the high-risk groups. (**F**). Comparison of the enrichment scores of 13 immune-related pathways between the low- and the high-risk groups. * *p* < 0.05, ** *p* < 0.01, *** *p* < 0.001.

As immune micro-environmental abnormality plays an important role in oncogenesis, invasion, and metastasis, to further explore the relationship between the protein-based prognostic signature and immunity, we further evaluated the tumor micro-environmental (TME) characteristics between different risk groups. The result of TME showed that the stromal score (*p* < 0.05), immune score (*p* < 0.05), and ESTIMATE score (*p* < 0.05) were higher in the high-risk group than in the low-risk group (Figure 5A). CD8^+^ T cells, plasma cells, and activated NK cells were significantly elevated in the low-risk group (*p* < 0.05). In contrast, resting dendritic cells, activated dendritic cells, and neutrophils were obviously increased in the high-risk group (*p* < 0.05) (Figure 5B). The protein-associated signature was positively correlated with resting dendritic cells and activated dendritic cells (*p* < 0.001), but negatively correlated with activated NK cells (*p* < 0.01), plasma cells (*p* = 0.01), and CD8^+^ T cells (*p* < 0.001, Figure 5C). Differential and correlated analyses demonstrated consistency in the immunologic characteristics. Weak to moderate correlations were observed among various tumor-infiltrating immune cells (Figure 5D). Significant differences were found in the enrichment scores of different immune cell subpopulations (Figure 5E) and associated functions or pathways (Figure 5F) using ssGSEA, including HLA and checkpoint, across different risk groups. Thus, the heterogeneity of immune cell infiltration in THCA could be a novel indicator with a potential clinical significance for immunotherapy.

### 3.6. Protein-Based Signature Is Associated with Immunization Checkpoint Block

**Figure 6:**
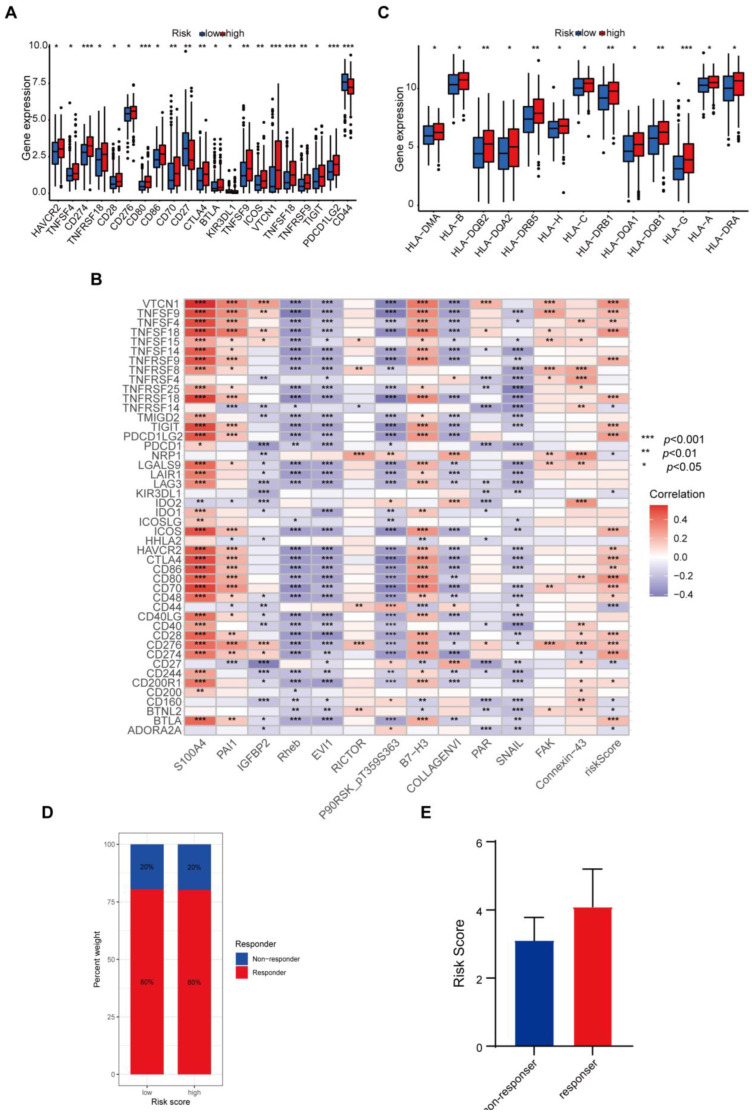
Protein-Based Signature is Associated with Immunization Checkpoint Block. (**A**) Comparison of the expression of immune checkpoints between the different risk groups. (**B**) Correlations with immune checkpoints. (**C**) The expression of the HLA family in the low- and high-risk groups. (**D**) The differences in response results to immunotherapy between the low- and high-risk groups in PTC patients. (**E**) The correlation between immunotherapy responsiveness and risk score in PTC patients. * *p* < 0.05, ** *p* < 0.01, *** *p* < 0.001.

Cancer cells must have evaded the anti-tumor immune response to grow progressively, which relies in part on the expression on their surface of proteins with immunosuppressive functions, such as programmed death ligand 1 (PD-L1) [20]. The ability of cancer cells to evade immune detection often leads to malignant progression and poor outcome in THCA patients. To understand how the protein-based signature affects the prognosis, we assessed the differences in immune checkpoint blocks (ICBs) in THCA, including the levels of immune checkpoint genes and HLA component expression. To avoid bias from different THCA histological types, we counted all types in the dataset and found four cases of nonencapsulated sclerosing carcinomas, one case of oxyphilic adenocarcinoma, one case of follicular carcinoma, and the remaining were papillary thyroid carcinomas (PTCs). Since the sample size of THCA types other than PTCs was too small to be representative, we only evaluated the tumor immune dysfunction and exclusion (TIDE) scores in PTCs. We found that in the high-risk group, levels of immune checkpoint genes like *HAVCR2*, *TNFSF4*, *CD274*, *TNFRSF18*, *CD28*, *CD276*, *CD80*, *CD86*, *CD70*, *CTLA4*, *BTLA*, *TNFSF9*, *ICOS*, *VTCN1*, *TNFSF18*, *TNFRSF9*, *TIGIT*, and *PDCD1LG2* were elevated, whereas *KIR3DL1* and *CD44* declined (Figure 6A). The correlation between immune checkpoint genes and risk scores also confirmed these results when we enrolled more immune checkpoint genes. Several genes in the signature including *Rheb*, *EVI1*, and *SNAIL*, were found to have negative correlations with nearly all checkpoints (Figure 6B). We further compared the differences in *HLA* expression. *HLA-A*, *HLA-B*, *HLA-C*, *HLA-DMA*, *HLA-DQA1*, *HLA-DQA2*, *HLA-DQB1*, *HLA-DQB2*, *HLA-DRA*, *HLA-DRB1*, *HLA-DRB5*, *HLA-G*, and *HLA-H* were evidently increased in the high-risk group (Figure 6C). Finally, we evaluated the potential immunotherapy response in each patient by the TIDE algorithm. However, the results showed that both PTC groups responded consistently to an immune checkpoint blockade with no difference (Figure 6D). The risk score tended to be higher in the PTC responder group than in the PTC non-responder group, yet not to a significant degree (Figure 6E). 

### 3.7. Mutational Landscape Based on the Protein-Associated Signature

To further elucidate the mechanism underlying tumorigenesis and progression in THCA patients, we compared the tumor mutational burden (TMB) across different risk groups based on somatic mutation. We found that the high-risk group had a significantly higher TMB than the low-risk group (*p* = 0.016) (Supplementary Figure S7A). Additionally, the mutational landscape revealed a marked increase in BRAF mutation in the high-risk group (Supplementary Figure S7B). The overall survival of the low-TMB group was better compared to the high-TMB group (*p* < 0.001) (Supplementary Figure S7C). In a stratified survival analysis, we examined the combined impact of the TMB and prognosis signature, which confirmed that the TMB would not affect the predictive power of the signature for prognosis (*p* < 0.001) (Supplementary Figure S7D). 

### 3.8. Potential Predictive Biomarker for Chemotherapy and Targeted Therapy

**Figure 7:**
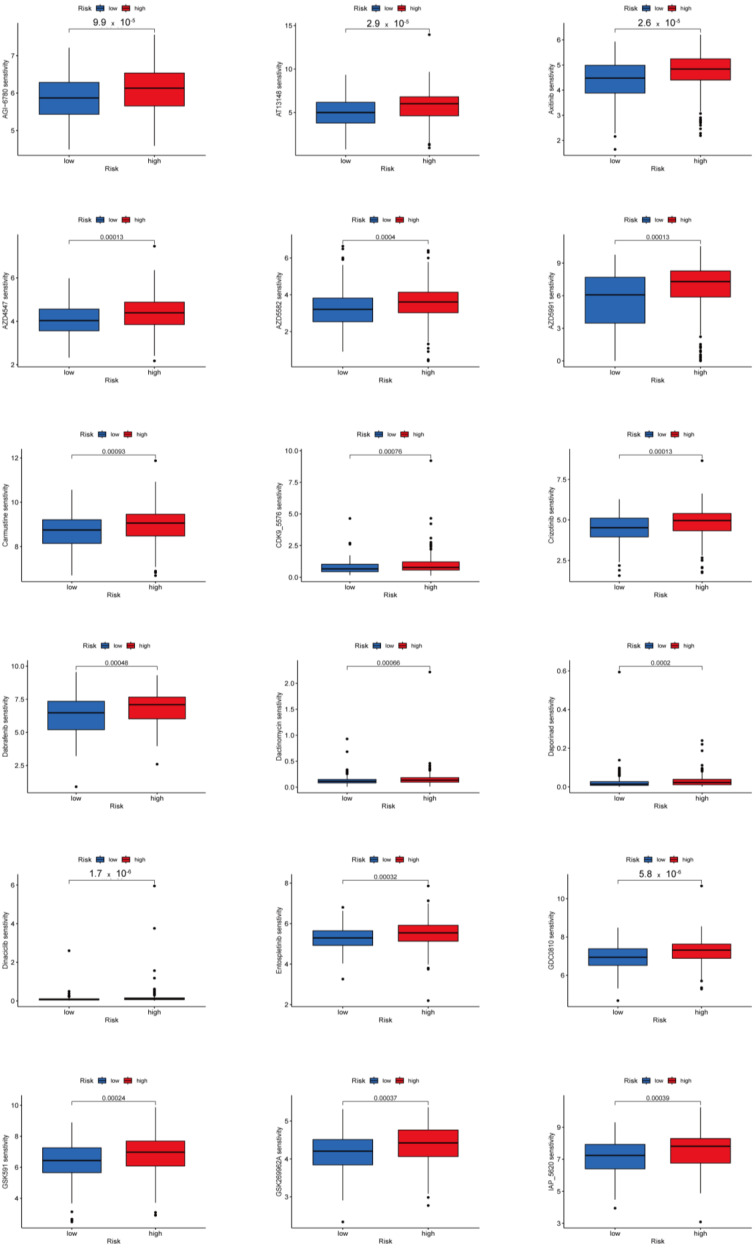
Potential Predictive Biomarker for Chemotherapy and Targeted Therapy. Patients with PTC in the high-risk group had higher estimated IC50 compared to those in the low-risk group.

We investigated potential predictive biomarkers for chemotherapy and targeted therapy in all PTC samples and found that the high-risk group had a higher sensitivity to AGI-6780, AT13148, AZD4547, AZD5582, AZD5991, GDC0810, GSK591, GSK269962A, I-BET-762, I-BRD9, KRAS inhibitor, LCL161, Mirin, OSI-027, Rapamycin, Dactinomycin, Carmustine, Zoledronate, Pevonedistat, Tozasertib, Uprosertib, Axitinib, Dabrafenib, Crizotinib, Entospletinib, and Ibrutinib than the low-risk group (*p* < 0.001) (Figure 7, Supplementary Figure S8). Therefore, the established protein-associated signature might aid in choosing effective chemotherapeutic agents and developing personalized target drugs for PTC.

## 4. Discussion

Thyroid carcinoma, as the most common endocrine system malignant tumor, poses a threat to health and well-being, especially in patients who cannot be operated or who have a recurrence after an operation and who have no response to iodine therapy [21,22]. The mechanisms of tumorigenesis and progression of THCA are still unclear. There is an urgent demand to establish a novel prognosis signature and clarify potential mechanisms in THCA. Although the application of next-generation sequencing makes the analysis of RNA and DNA levels popular in oncology research, it should be noted that the majority of genes play their roles at the protein level. The development of proteomic technology and bioinformatics has provided new insights for comprehensive exploration of the molecular mechanisms of cancer pathogenesis and valuable information for prognosis and treatment decisions [23,24,25,26,27]. However, to date, there are relatively few studies on the role of proteomic models in THCA patients.

In our study, we randomly divided the entire set into a training and a testing set, and developed the model using the training set. After univariate Cox analysis and LASSO Cox regression analysis, we created a prognostic model composed of 13 proteins. Patients in the high-risk group showed significantly poor OS. The candidate proteins involved in the prognostic signature have been proven to engage in cancer development. S100A4 is a small calcium-binding protein that can affect multiple biological processes on its binding partners [28,29]. S100A4 is associated with poor survival in THCA as well as in glioma [30], bladder [31], pancreatic [32], and breast cancers [33]. PAI1 (SERPINE1) can not only inhibit caspase 3 [34], but also inhibit the cleavage of FasL and its abscission by the plasmin on the cell surface [35] to resist tumor cell apoptosis. IGFBP2 promotes tumor progression by inducing the alternative polarization of macrophages through the STAT3 pathway [36]. Emerging evidence has demonstrated that COL6A1 has diverse biological functions, including cell migration, differentiation, embryonic development, and maintenance of cell stemness in human malignancies [37]. Protease-activated receptors (PARs) are a unique family of G-protein coupled receptors, which play important roles in promoting cancer metastasis [38,39]. It has been widely reported that Snail played crucial roles in hepatocarcinogenesis and metastasis [40]. Several lines of evidence have indicated that the gap junction protein connexin 43 (Cx43) controls the response of glioblastoma to temozolomide (TMZ) [41], and blocking Cx43 using different approaches restores TMZ sensitivity [42,43]. Rictor correlates with poor prognosis of gastric cancer patients mainly through sensing growth factor concentration, regulating cell proliferation, survival, metabolism, and cytoskeletal remodeling [44]. In addition, our results suggest that B7-H3 was associated with increased risk in THCA patients, which was inconsistent with a previous report [45]. FAK expression is crucial for the development and growth of thyroid tumors. Recent research indicates that periostin, derived from cancer-associated fibroblasts, enhances thyroid tumor growth by activating FAK-STAT3 signaling [46]. Rheb is an important positive regulator of the mTORC1 pathway. Recent research indicates that Rheb degradation and subsequent mTORC1 inactivation play roles in cancer cell survival under glucose deprivation [47]. To date, the prognostic impact of EVI1 has not been clearly defined in real-world datasets. Some studies have found an association between high EVI1 expression and improved survival rates [48,49], which is consistent with our findings. High levels of phosphorylated p90RSK expression in ER-positive breast cancer tissues were linked to tumor shrinkage and decreased tumor volume following surgery. This association was particularly significant in ER-positive tumors [50]. The accuracy of the signature prediction for 1, 3, and 5 years of the three sets was more than 0.7. The combination of multiple proteins provided superior predictive efficiency compared to a single biomarker. The protein signature outperformed previously published signatures in predicting the prognosis of THCA [22,51,52]. Subgroup survival analysis, incorporating the clinical features, found that a high risk was associated with poorer prognosis in age > 65, female, T3–4 stage, N1 stage, M0 stage, and Stage III–IV subgroups. Additionally, multi-ROC curve analyses and the evaluation of the nomogram support the potential clinical application of this signature for prognosis prediction.

Expression analysis revealed that 245 proteins were differently expressed between the two risk groups. The results of GO and KEGG revealed that these differently expressed proteins were associated with the regulation of cell–cell adhesion, chromosomal region, PI3K binding, regulation of DNA metabolic process, EGFR tyrosine kinase inhibitor resistance, FoxO signaling pathway, NF-κB signaling pathway, PD-L1 expression, and PD-1 checkpoint pathway in cancer. Interestingly, these pathways were engaged in the progression of cancer. During malignant progression, epithelial cancer cells dissolve their cell–cell adhesion and gain invasive features [53]. Chromosomal rearrangements often result in active regulatory regions juxtaposed upstream of an oncogene to generate an expressed gene fusion [54]. PI3Ks are enzymes that belong to a family of plasma membrane-associated lipid kinases [55], implicated in the pathogenesis of many cancers [56]. NF-κB regulates the expression of genes involved in many processes that play a key role in the development and progression of cancer such as proliferation, migration, and apoptosis [57]. Epidermal growth factor receptor (EGFR), one of the most studied receptor kinases, is a drug target for cancer therapy, because its kinase activity correlates with tumorigenicity [58]. Emerging evidence has revealed that the expression of PD-L1 on tumor cells leads to immunosuppression and consequently enhances aggressiveness [59]. Recent studies have reported that PD-L1 is highly expressed in a subset of patients with advanced thyroid cancer and correlates with a higher risk of recurrence and shortened disease-free survival [60,61]. These differences may contribute to the different prognosis between the two groups.

The tumor immune microenvironment plays a key role in regulating the processes of tumor development, invasion, and metastasis [62]. To gain deeper insights into the immune cell infiltration in THCA, we explored the relationship between the protein-based prognostic signature and immune cell infiltration. We found that resting dendritic cells, activated dendritic cells, and neutrophils were significantly elevated in the high-risk group, while CD8^+^ T cells, plasma cells, and activated NK cells were remarkably elevated in the low-risk group. CD8^+^ T cells are typically regarded as a uniform population of cells that secrete large amounts of IFN-γ and the protease granzyme B, which act synergistically to kill infected or tumorigenic cells [63]. Plasma cells play a beneficial role in the majority of cancer types [64], suggesting the low-risk group THCA patients benefit from the infiltrating plasma cells. There is emerging evidence of increased NK cell-mediated tumor cell killing [65]. In THCA patients, tumor-associated neutrophils, which are capable of promoting tumor progression by secreting a wide range of cytokines [66], correlated with a larger tumor size [67]. Several studies have shown that tumor-infiltrating dendritic cells are related to more advanced tumor T stage and lymph node metastasis in THCA [68,69]. Our results indicate increased anti-tumor immune activity in the low-risk group, partially explaining the predictive value of the prognostic signature. The immune cell subpopulation enrichment analysis and the ssGSEA-related functions or pathway analysis revealed that scores for nearly all the items were higher in the high-risk group, which indicated that THCA patients in the high-risk group seemed to be more immunogenic. Moreover, the high-risk group had a higher immune score, stromal score, and ESTIMATE score, which further suggests that patients with a high-risk score appeared to be more immunogenic and might benefit from immunotherapy.

We further evaluated the differences in the ICB between the two groups. The high-risk groups featured higher levels of HLA family genes and higher levels of immune checkpoint genes. Lymphocyte-activation gene 3 (LAG3) is an immune inhibitory checkpoint and is expressed on the surface of lymphocytes [70], such as CD4+ T cells, CD8+ T cells, natural killer (NK) cells, NK T (NKT) cells, and regulatory T (Treg) cells. LAG3 inhibits the tumor immune microenvironment by accelerating T cell exhaustion and blocking T cell proliferation [71]. Programmed death-ligand 1 (PD-L1, CD274), an immune checkpoint protein expressed on cancer cells and immune cells, interacts with its binding partner programmed cell death-1 (PD-1) to inhibit T cell proliferation and cytokine production [72]. TIGIT in CD4+ T cells induce immunosuppression through inhibiting T cell proliferation directly by inducing the down-expression of T-bet, GATA3, IRF4, and RORc, which reduce the level of pro-inflammatory IFN-γ while increasing the level of anti-inflammatory IL-10 [73]. PDCD1LG2 (PD-L2) is a second ligand for PD-1 and inhibits T cell activation [74]. The immune inhibitory checkpoint contributed to the worse survival in the high-risk groups. On the other hand, MHC molecules were found to be overexpressed in the high-risk groups. Tumor recognition by immune cells requires presentation of non-self peptides (neoantigens) by tumor cells through MHC Class I or II complexes. Loss or reduced expression of MHC or their subunits abrogates T cell-mediated anti-tumor immunity [75]. Tumor-specific MHC-II is associated with good outcomes in cancer patients, including those with immunotherapies [76]. The higher expression of MHC molecules in THCA patients of the high-risk group may explain the better prognosis of THCA than other tumors. Our results suggest that patients in the high-risk group have a higher ICB, which may have a possible good anti-tumor immune response. 

To delve into the cause of tumorigenesis, the TMB of two groups was compared and a higher TMB was found in the high-risk group. In our present study, the most frequent mutation was the BRAF missense mutation. Previous studies have found the BRAF T1799A mutation in approximately 45% of PTC and 25% of apparently PTC-derived anaplastic thyroid cancers, but not in follicular thyroid cancer (FTC) and benign thyroid tumors [77]. BRAF T1799A mutation contributes to poor clinicopathologic outcomes of PTC [78] and this mutation is often correlated with a loss of radiotherapy response and recurrence of PTC [77]. The BRAF V600E mutant has increased kinase activity, resulting in promoting cell growth, differentiation, and survival [79]. Kaplan–Meier analysis shows that the low-TMB group had better OS than the high-TMB group; in addition, the combined Kaplan–Meier analysis demonstrated that the risk score was a reliable predictive indicator independent of the TMB. Patients with a high TMB have been reported to have a more sensitive response to immune checkpoint blockade, such as anti-PD-1 agents [80]. The risk score tended to be higher in the ICB responder group than in the non-responder group, although not to a significant degree.

Effectiveness of chemotherapy and targeted treatment between the two risk groups were predicted by the GDSC dataset. In the high-risk group, the THCA patients were more sensitive to AGI-6780, AT13148, AZD4547, AZD5582, AZD5991, GDC0810, GSK591, GSK269962A, I-BET-762, I-BRD9, KRAS inhibitor, LCL161, Mirin, OSI-027, Rapamycin, Dactinomycin, Carmustine, Zoledronate, Pevonedistat, Tozasertib, Uprosertib, Axitinib, Dabrafenib, Crizotinib, Entospletinib, and Ibrutinib, which might be a potential treatment option for THCA. Our results suggest that THCA patients in the high-risk groups had more ICB expressions, a higher TMB, and PTC patients in the high-risk groups responded more to sensitivity to chemotherapy and targeted treatment.

However, this study has some limitations that should be considered. To begin with, it is a preliminary study based on bioinformatics tools, incorporating real-world samples; other cohort validation will allow for a more rigorous design. In addition, our molecular results lack experimental evidence in vivo and in vitro.

## 5. Conclusions

In summary, we constructed a valid prognostic protein signature to predict outcomes for THCA patients, demonstrating a strong predictive ability. We also evaluated the differences in immunotherapy response and chemotherapeutic drug sensitivity between the two risk groups. These results could advance our understanding of THCA pathogenesis and provide new strategies for personalized therapy.

## Figures and Tables

**Table 1:** Proteins used for the construction of risk prognostic model.

ID	Coefficient	HR	HR.95L	HR.95H	*p* Value
S100A4	0.190685583465921	2.269585	1.430698	3.600352	0.000499
PAI1	0.640128590811076	5.331818	2.091542	13.59202	0.000456
IGFBP2	2.48571958440727	5.234716	1.352732	20.25697	0.016504
Rheb	−3.09616014265545	0.13992	0.043317	0.451963	0.001011
EVI1	−1.96032334732288	0.021668	0.000912	0.514674	0.017743
RICTOR	−1.7411650797503	1.857881	1.070301	3.225001	0.027707
P90RSK_pT359S363	−6.6880866540024	0.095157	0.012577	0.719942	0.022714
B7-H3	−0.220113199086768	5.247871	1.703994	16.16211	0.003869
COLLAGENVI	1.84909089061224	2.665753	1.269488	5.597718	0.009588
PAR	1.0667455542712	3.813442	1.765558	8.236686	0.000657
SNAIL	10.6429840082611	39.17723	1.594989	962.2985	0.024717
FAK	−3.09948996444977	4.461859	1.158865	17.17904	0.029681
Connexin-43	0.941781881570662	2.214479	1.017812	4.818098	0.045019

## Data Availability

The protein expression data of THCA patients were freely down-loaded from the TCPA database (https://tcpaportal.org/tcpa/ (accessed on 18 July 2022)). The clinical information of these patients was obtained from the TCGA database (https://gdc.cancer.gov/ (accessed on 18 July 2022)).

## Supplementary Materials

The following supporting information can be downloaded at: https://www.mdpi.com/article/10.3390/biomedicines12092066/s1, Figure S1: Overall survival based on the prognostic associated proteins; Figure S2: Principal component analysis based on the expression level of 13 prognostic proteins; Figure S3: Univariate and multivariate Cox regression analysis; Figure S4: Stratified analysis; Figure S5: Functional Enrichment Analysis Based on Risk Model; Figure S6: Protein-protein interaction (PPI) network; Figure S7: Mutational Landscape Based on the Protein-Associated Signature; Figure S8: Potential Predictive Biomarker for Chemotherapy and Targeted Therapy.
