# Supplementary figures and images for: Prognostic Protein Biomarker Screening for Thyroid Carcinoma Based on Cancer Proteomics Profiles
Source: Biomedicines. 2024 Sep 10;12(9):2066. doi: 10.3390/biomedicines12092066 (PMC11428938; doi:10.3390/biomedicines12092066)

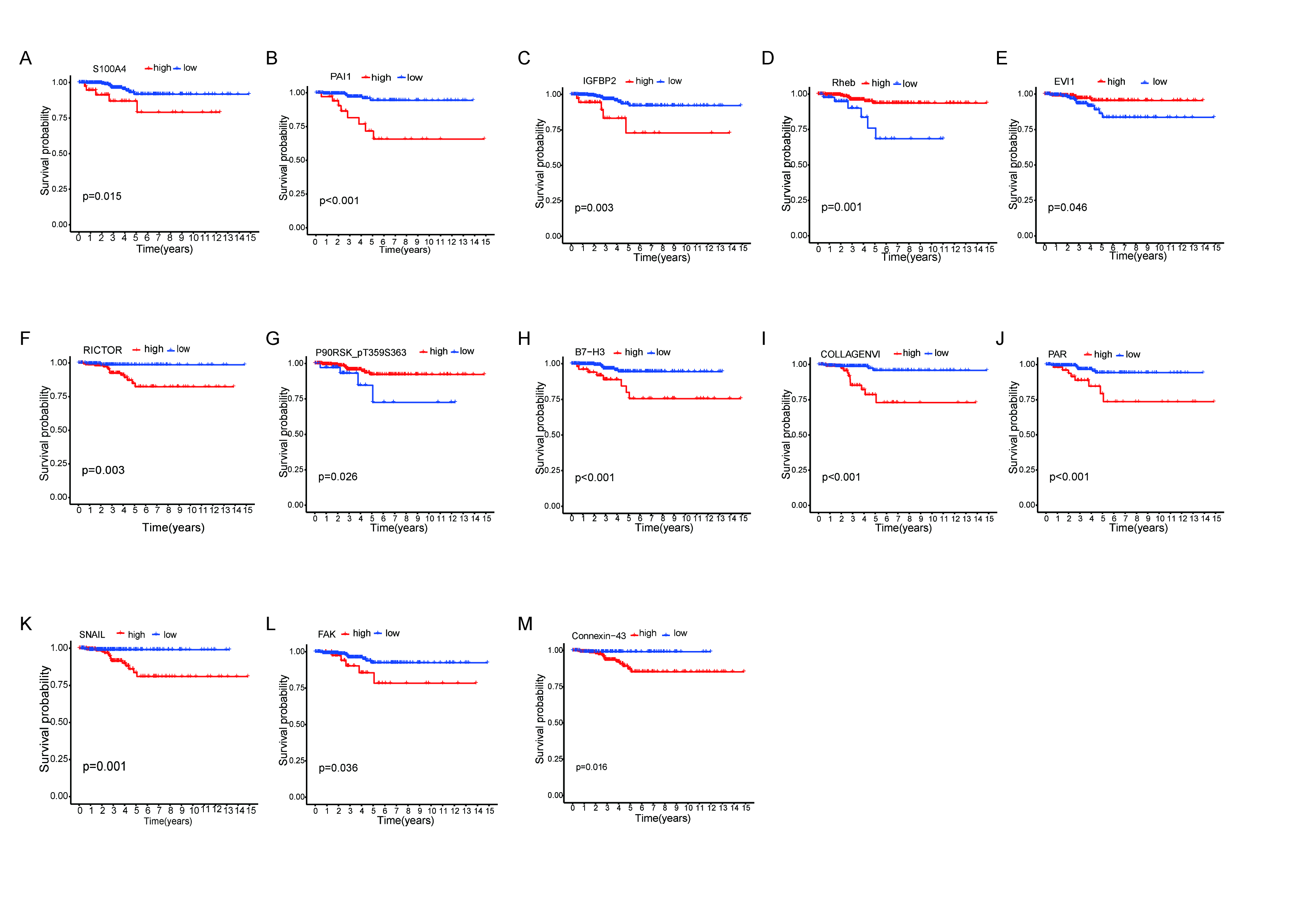

Supplement: Supplementary file 1 [file biomedicines-12-02066-s001.zip › Supplementary Figure S1.tif]

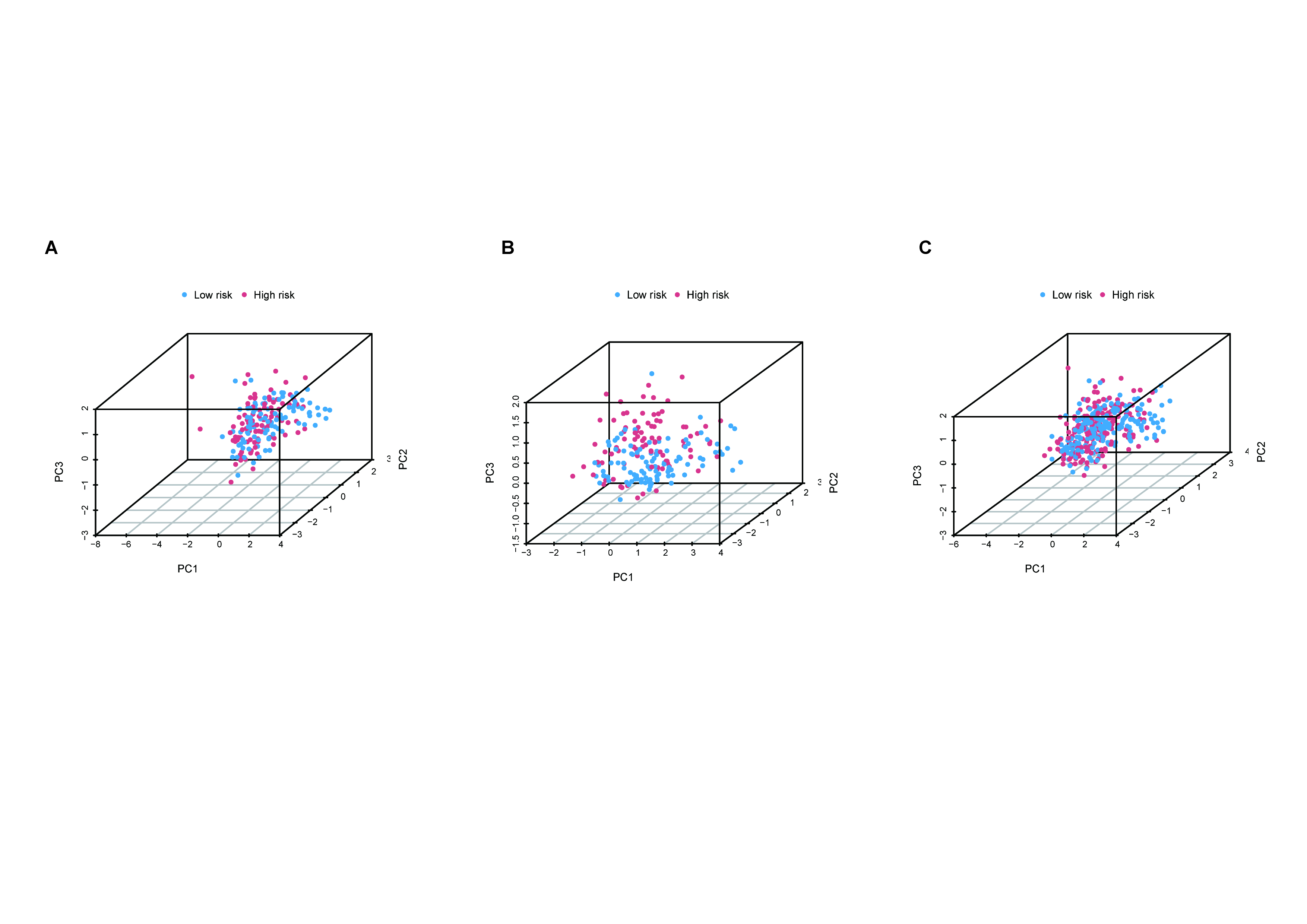

Supplement: Supplementary file 1 [file biomedicines-12-02066-s001.zip › Supplementary Figure S2.tif]

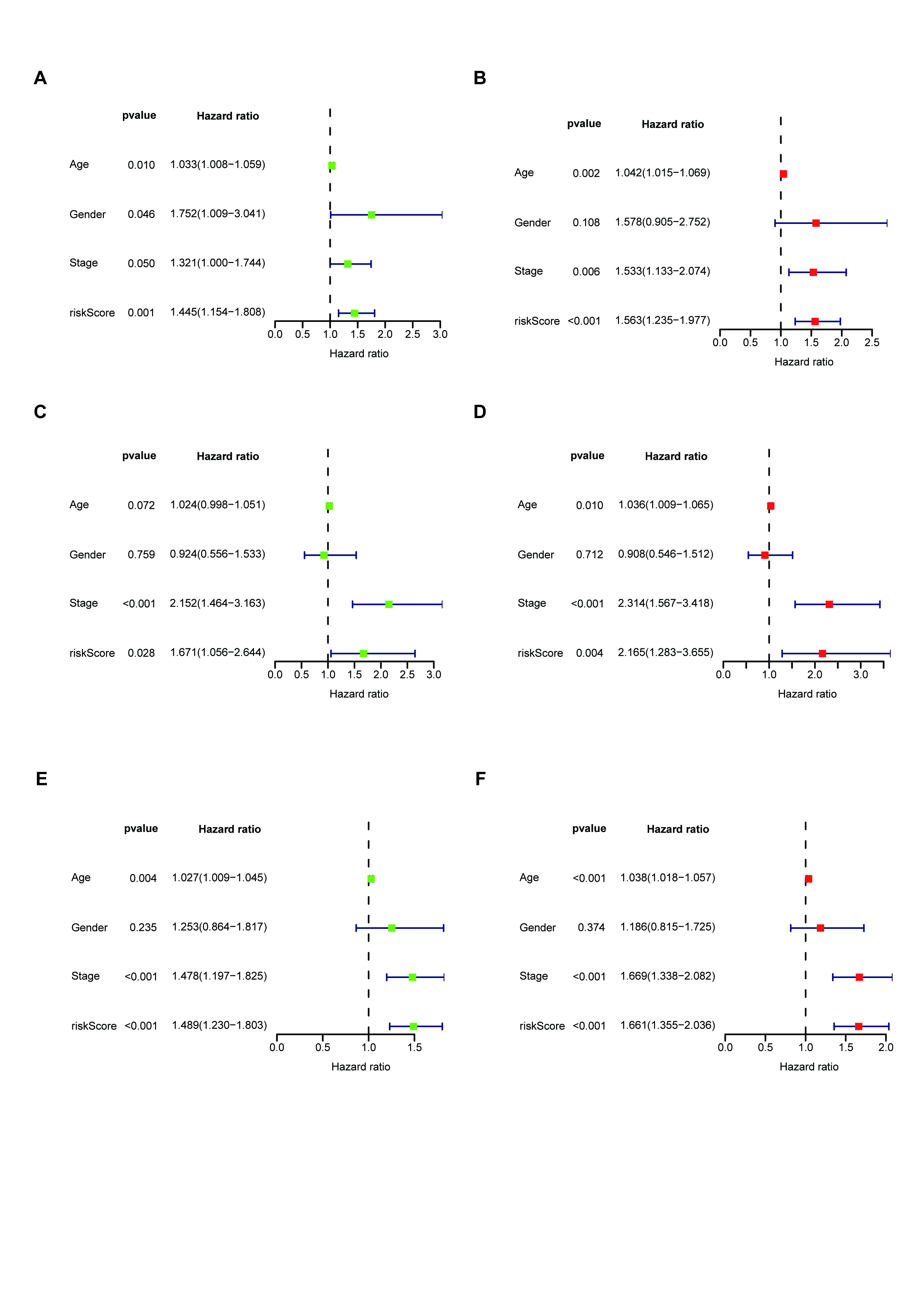

Supplement: Supplementary file 1 [file biomedicines-12-02066-s001.zip › Supplementary Figure S3.tif]

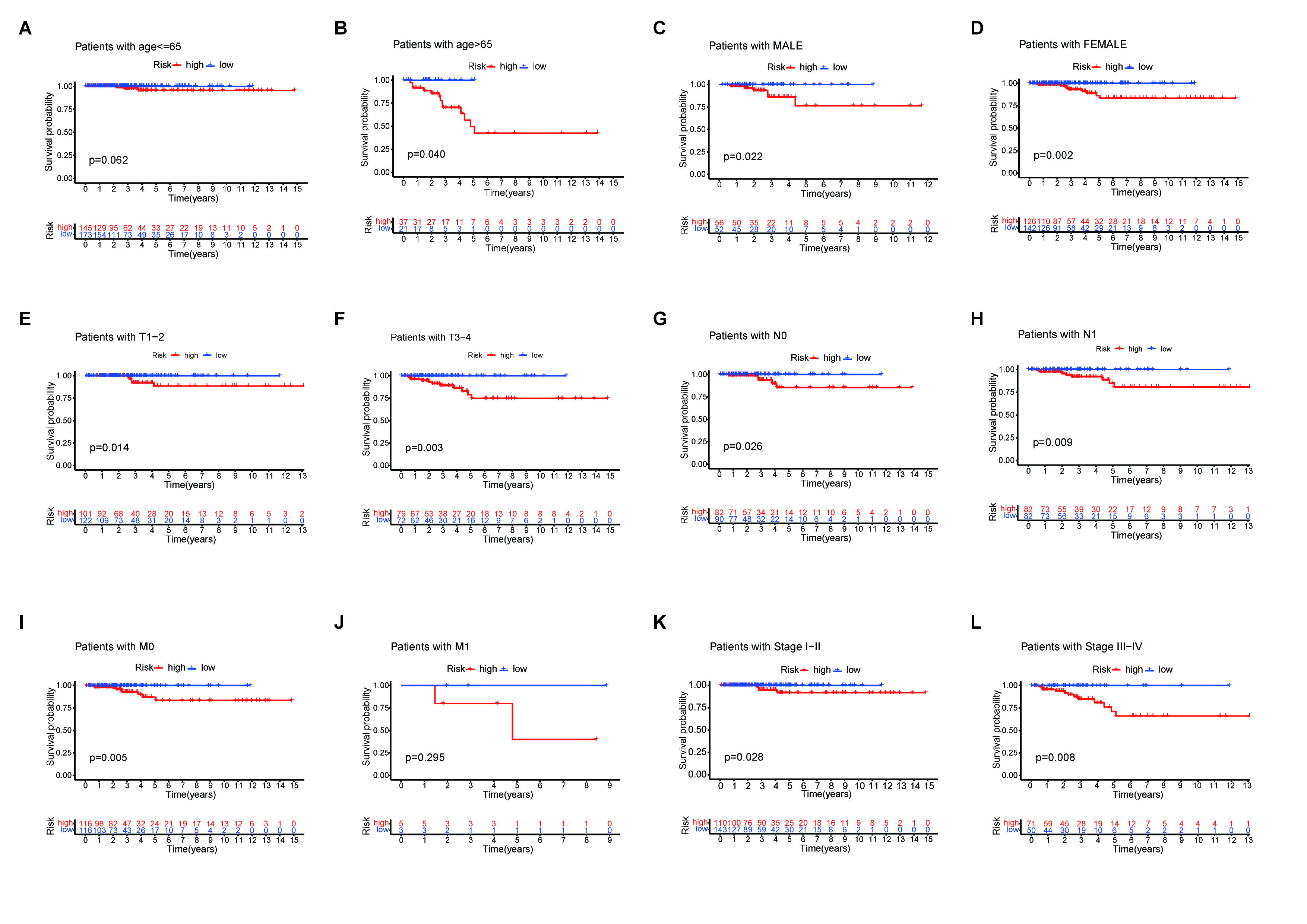

Supplement: Supplementary file 1 [file biomedicines-12-02066-s001.zip › Supplementary Figure S4.tif]

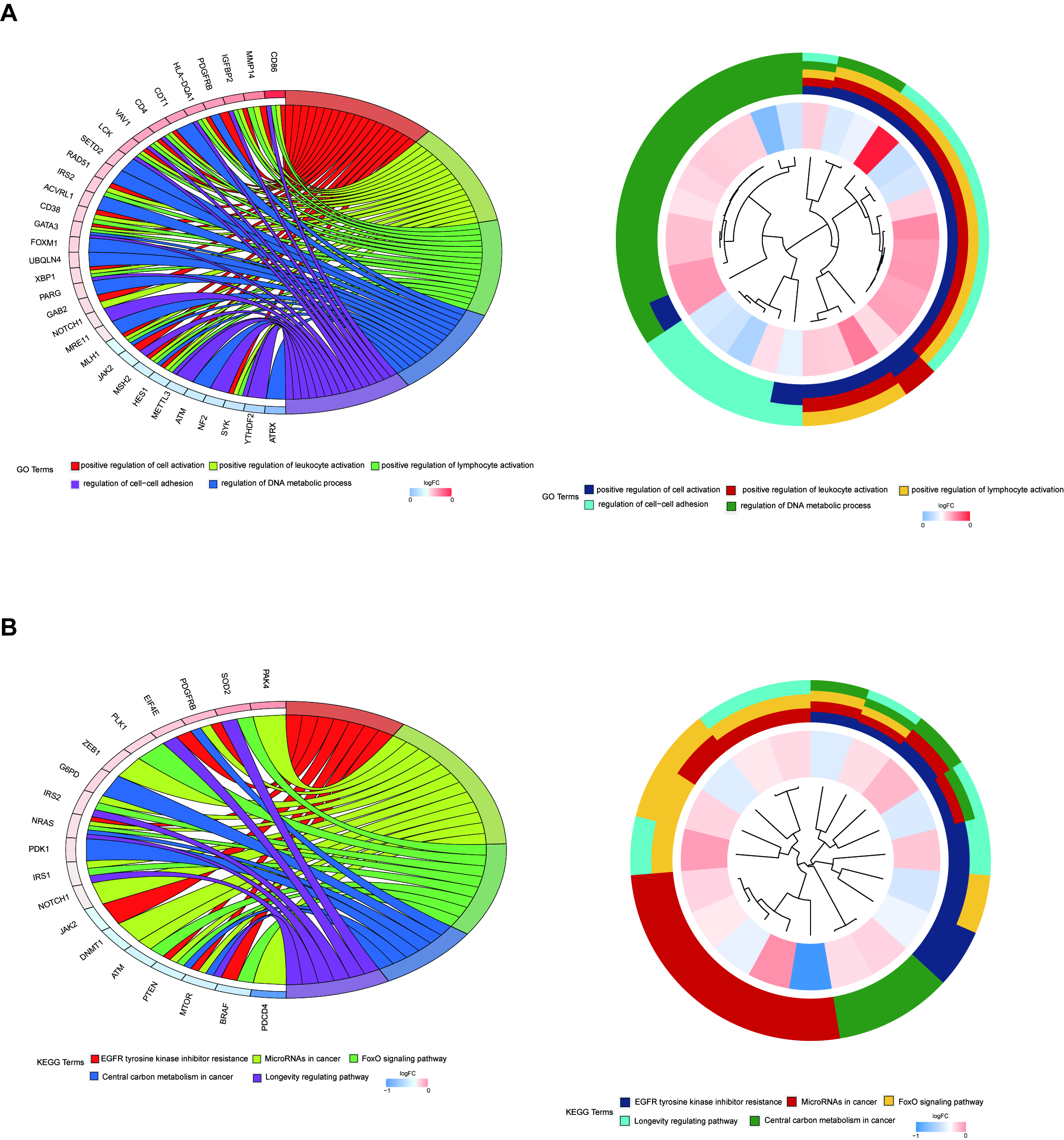

Supplement: Supplementary file 1 [file biomedicines-12-02066-s001.zip › Supplementary Figure S5.tif]

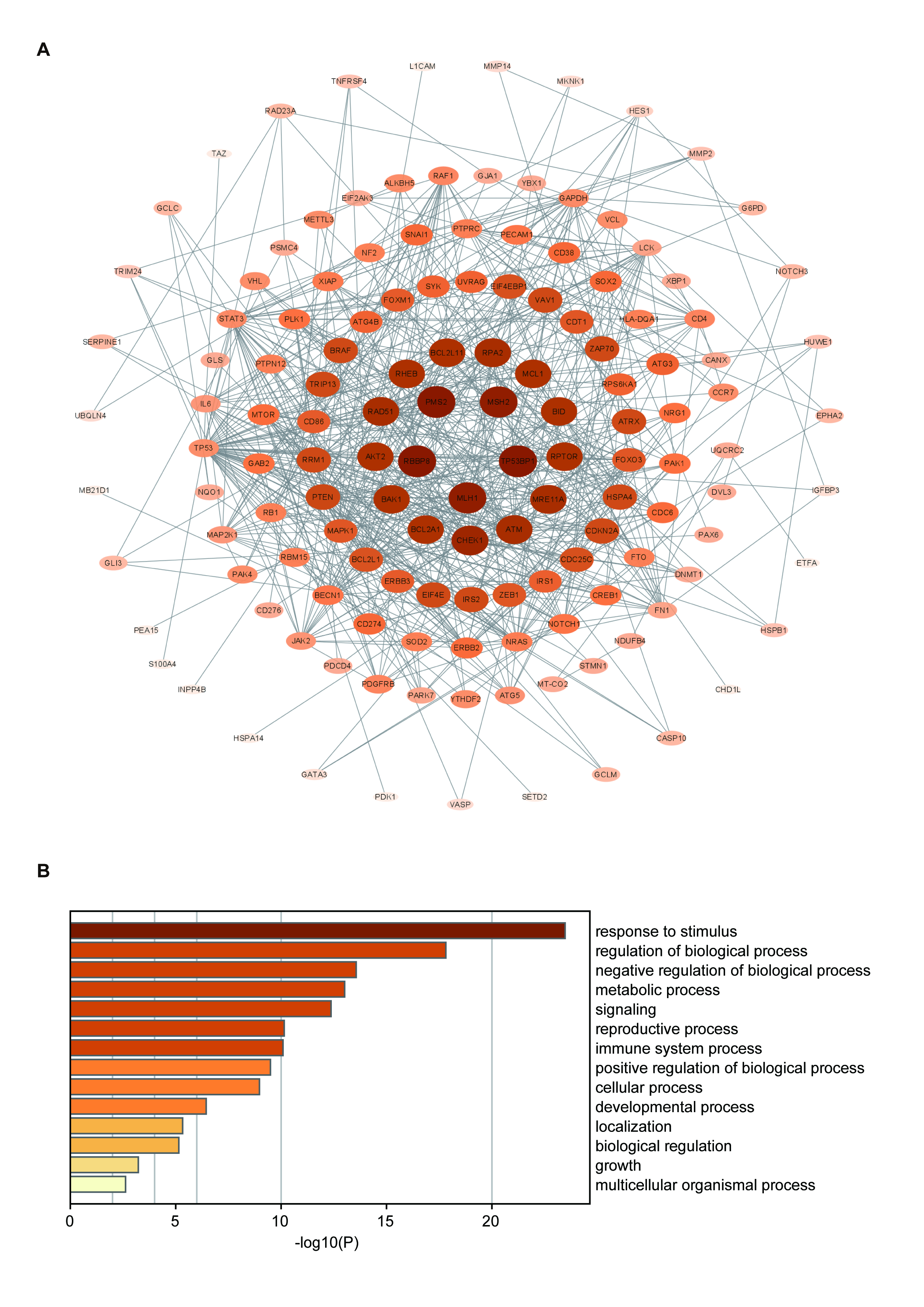

Supplement: Supplementary file 1 [file biomedicines-12-02066-s001.zip › Supplementary Figure S6.tif]

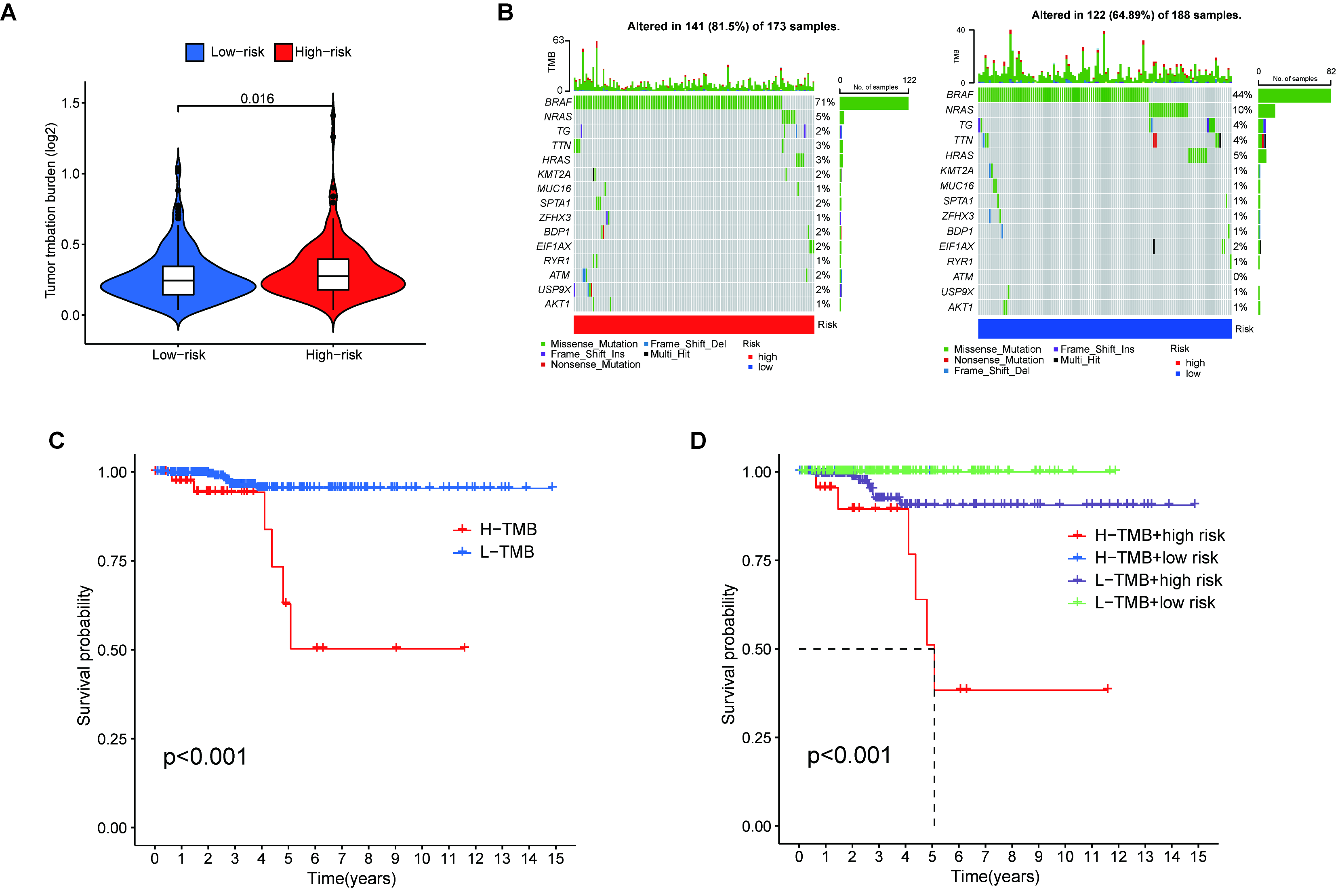

Supplement: Supplementary file 1 [file biomedicines-12-02066-s001.zip › Supplementary Figure S7.tif]

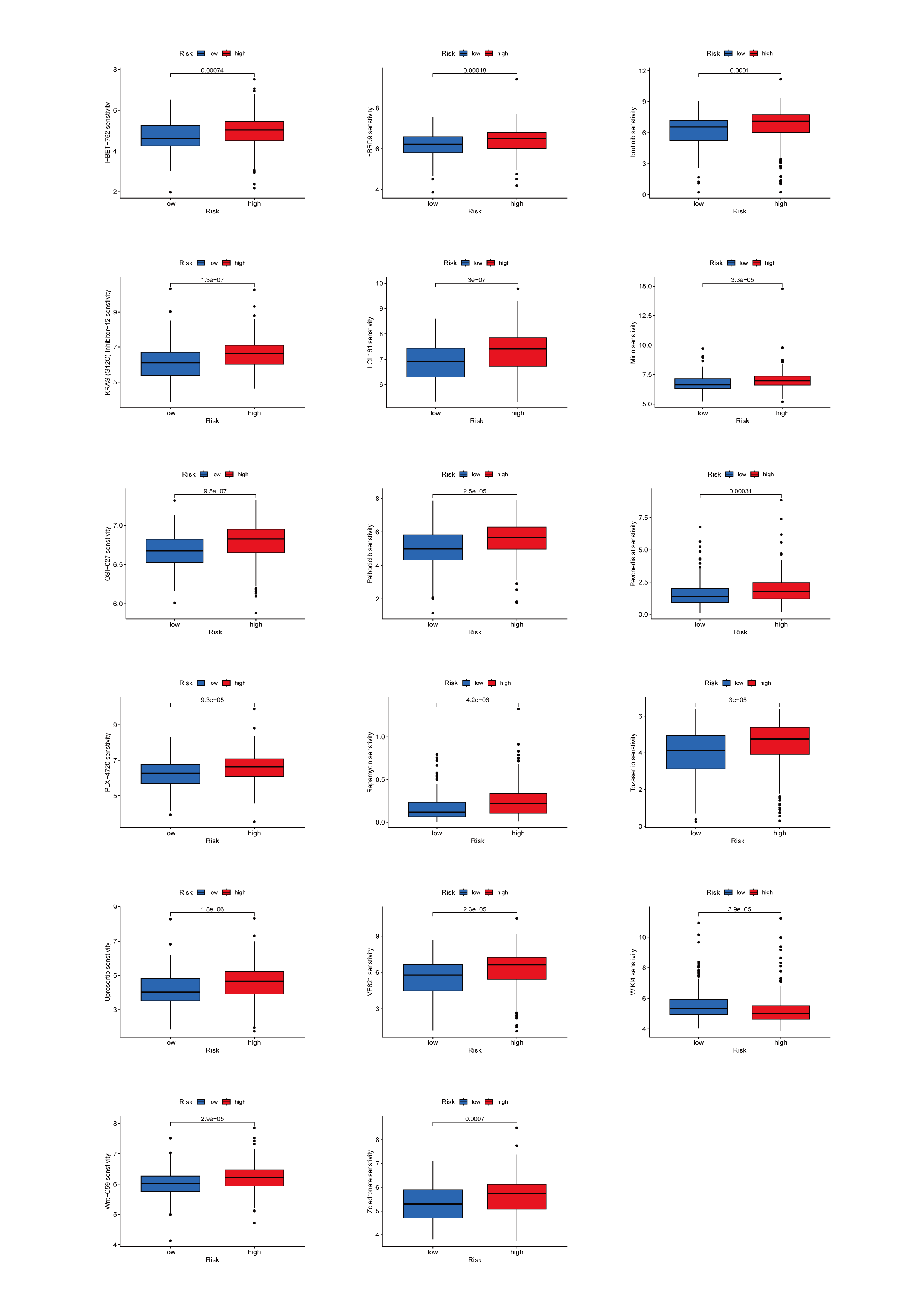

Supplement: Supplementary file 1 [file biomedicines-12-02066-s001.zip › Supplementary Figure S8.tif]
